# Resistance Patterns Selected by Nevirapine vs. Efavirenz in HIV-Infected Patients Failing First-Line Antiretroviral Treatment: A Bayesian Analysis

**DOI:** 10.1371/journal.pone.0027427

**Published:** 2011-11-23

**Authors:** Nicole Ngo-Giang-Huong, Gonzague Jourdain, Billy Amzal, Pensiriwan Sang-a-gad, Rittha Lertkoonalak, Naree Eiamsirikit, Somboon Tansuphasawasdikul, Yuwadee Buranawanitchakorn, Naruepon Yutthakasemsunt, Sripetcharat Mekviwattanawong, Kenneth McIntosh, Marc Lallemant, for the Program for HIV Prevention and Treatment (PHPT) study group

**Affiliations:** 1 Institut de Recherche pour le Développement (IRD) UMI 174 - PHPT, Marseilles, France; 2 Department of Medical Technology, Faculty of Associated Medical Sciences, Chiang Mai University, Chiang Mai, Thailand; 3 Department of Immunology and Infectious Diseases, Harvard School of Public Health, Boston, Massachusetts, United States of America; 4 Ratchaburi Hospital, Ratchaburi, Thailand; 5 Maharat Nakonratchasima Hospital, Nakonratchasima, Thailand; 6 Samutprakarn Hospital, Samutprakarn, Thailand; 7 Buddhachinaraj Hospital, Pitsanuloke, Thailand; 8 Chiang Kham Hospital, Chiang Kham, Thailand; 9 Nong Khai Hospital, Nong Khai, Thailand; 10 Pranangklao Hospital, Bangkok, Thailand; 11 Division of Infectious Diseases, Children's Hospital, Boston, Massachusetts, United States of America; Massachusetts General Hospital, United States of America

## Abstract

**Background:**

WHO recommends starting therapy with a non-nucleoside reverse transcriptase inhibitor (NNRTI) and two nucleoside reverse transcriptase inhibitors (NRTIs), i.e. nevirapine or efavirenz, with lamivudine or emtricitabine, plus zidovudine or tenofovir. Few studies have compared resistance patterns induced by efavirenz and nevirapine in patients infected with the CRF01_AE Southeast Asian HIV-subtype. We compared patterns of NNRTI- and NRTI-associated mutations in Thai adults failing first-line nevirapine- and efavirenz -based combinations, using Bayesian statistics to optimize use of data.

**Methods and Findings:**

In a treatment cohort of HIV-infected adults on NNRTI-based regimens, 119 experienced virologic failure (>500 copies/mL), with resistance mutations detected by consensus sequencing. Mutations were analyzed in relation to demographic, clinical, and laboratory variables at time of genotyping. The Geno2Pheno system was used to evaluate second-line drug options. Eighty-nine subjects were on nevirapine and 30 on efavirenz. The NRTI backbone consisted of lamivudine or emtricitabine plus either zidovudine (37), stavudine (65), or tenofovir (19). The K103N mutation was detected in 83% of patients on efavirenz vs. 28% on nevirapine, whereas Y181C was detected in 56% on nevirapine vs. 20% efavirenz. M184V was more common with nevirapine (87%) than efavirenz (63%). Nevirapine favored TAM-2 resistance pathways whereas efavirenz selected both TAM-2 and TAM-1 pathways. Emergence of TAM-2 mutations increased with the duration of virologic replication (OR 1.25–1.87 per month increment). In zidovudine-containing regimens, the overall risk of resistance across all drugs was lower with nevirapine than with efavirenz, whereas in tenofovir-containing regimen the opposite was true.

**Conclusions:**

TAM-2 was the major NRTI resistance pathway for CRF01_AE, particularly with nevirapine; it appeared late after virological failure. In patients who failed, there appeared to be more second-line drug options when zidovudine was combined with nevirapine or tenofovir with efavirenz than with alternative combinations.

## Introduction

The World Health Organization (WHO) currently recommends starting antiretroviral (ARV) combination regimens with a non-nucleoside reverse transcriptase inhibitor (NNRTI) and two nucleoside reverse transcriptase inhibitors (NRTIs), i.e. nevirapine (NVP) or efavirenz (EFV), with lamivudine (3TC) or emtricitabine (FTC), plus zidovudine (ZDV) or tenofovir (TDF) [1]. The combination most commonly used in resource limited countries is a fixed dose formulation containing nevirapine, lamivudine and either stavudine (d4T) or zidovudine, and efficacy and drug failure are monitored for most subjects by clinical or, if available, CD4 criteria. Maintaining a failing first line regimen which includes two drugs with low genetic barriers to resistance, such as nevirapine or efavirenz, plus lamivudine as one of the NRTI's, poses a risk of accumulation of resistance mutations. This can, in turn, limit therapeutic drug options for the second-line therapies [2], [3], [4], [5], [6], [7], [8], [9].

In addition the pattern of drug-resistant mutations may differ according to the particular drug combinations used and the circulating HIV-1 subtypes. Although a large data base analysis comparing the NNRTI resistance patterns induced by efavirenz and nevirapine was recently published [10], there have been few studies performed in homogeneous groups of patients [11]. With regard to subtype, in subjects infected with HIV-1 subtype B, the thymidine analogue mutations pathway 1 or TAM-1 (including mutations M41L, L210W and T215Y) is probably more frequent than the TAM-2 pathway (including mutations D67N, K70R, T215F and K219E/Q) [12], [13], [14], although systematic studies of these pathways have not been done. In subtype C virus, Novitsky and colleagues [15] reported a distinct TAM pathway in patients failing ZDV/ddI-containing HAART.

Similarly, there may be different pathways for NVP or EFV resistance mutations which may impact on the success of second generation NNRTIs. The predominant subtype in Thailand is CRF01_AE, and there are few published studies analyzing the resistance mutation patterns that develop during virologic failure in this important subtype, prevalent throughout East and South-east Asia [8], [16], [17], [18].

Nationwide access to antiretroviral treatment in Thailand began in 2002, with gradually increasing coverage to more than 200,000 HIV-infected patients receiving combination antiretroviral drugs, usually beginning with one of the locally manufactured fixed-dose combinations, (d4T or ZDV)+3TC+NVP [19]. In case of toxicity, NVP is replaced by EFV.

The primary objective of this study was to describe and compare the patterns and frequencies of NNRTI and NRTI-associated mutations emerging on nevirapine- and efavirenz-based HAART in Thai HIV-infected adults failing their first-line treatment using Bayesian statistical methods, with a view toward supporting decisions regarding subsequent salvage treatment choices. Secondary objectives were to assess factors associated with more frequent occurrence of NNRTI and NRTI resistance mutations and to compare clusters of mutations observed under nevirapine and efavirenz at failure.

## Results

### Patient characteristics

A total of 138 subjects with virologic failure were identified, 19 of whom (13%) showed neither NNRTI nor NRTI mutations and were assumed to be non-compliant with their treatment. These 19 were not considered further in this analysis. Of 98 remaining subjects who initiated a first line nevirapine-based HAART, 10 had nevirapine replaced by efavirenz within 2–4 weeks for toxicity reasons. Of 21 subjects who initiated efavirenz-based HAART, 1 had efavirenz replaced by nevirapine. Thus, 89 subjects were on nevirapine- and 30 subjects on efavirenz-based HAART at the time of virologic failure and showed at least one resistance mutation. Their demographic, clinical (including NRTI backbone) and laboratory data at the time of genotyping are described in Table 1. The estimated length of time from HAART initiation to virologic failure was about. 220 days and the duration of failure before genotypic resistance testing was about 90 days; these two intervals were similar between the 2 groups. D4T and 3TC were more often used with nevirapine (P<0.001 and P<0.001) while ZDV, TDF and FTC were more used with efavirenz (P = 0.006, P = 0.007 and P = 0.001), which supports the need for statistical adjustments with respect to the NRTI backbone used.

**Table 1:** Comparative summary statistics for demographic, clinical and laboratory data.

	Nevirapine-based HAART (n = 89)	Efavirenz-based HAART (n = 30)	P-value
Age in years	31.4 (28.3–36.5)	32.6 (28.9–37.7)	0.258
Female	81 (91%)	25 (83%)	0.200
CDC stage B or C	30 (34%)(n = 88)	13 (46%)(n = 28)	0.267
CD4 count (cells/mm3)	241 (173–359)	159 (110–265)	0.005
HIV RNA (log10 copies/mL)	3.70 (3.28–4.09)	3.99 (3.75–4.69)	0.007
CRF01_AE	84 (94%)	29 (97%)	
Exposure to single-dose nevirapine	58 (81%)	10 (56%)	0.036
Time (days) from single-dose nevirapine to HAART initiation	139 (64–397)	387 (125–505)	0.299
ZDV backbone	20(22%)	15(50%)	0.006
d4T backbone	60(67%)	5(17%)	<0.001
TDF backbone	9(10%)	10(33%)	0.007
3TC backbone	82(92%)	19(63%)	<0.001
FTC backbone	7(8%)	10(33%)	0.001
Estimated duration (days) of virologic failure before HIV resistance genotype testing	91 (42–189)	93 (56–196)	0.743
Time (days) from HAART initiation to virologic failure	238 (112–609)	212 (88–441)	0.530

All figures are medians (interquartile ranges) or number (percent).

### Pattern of resistance mutations

**Figure 1:**
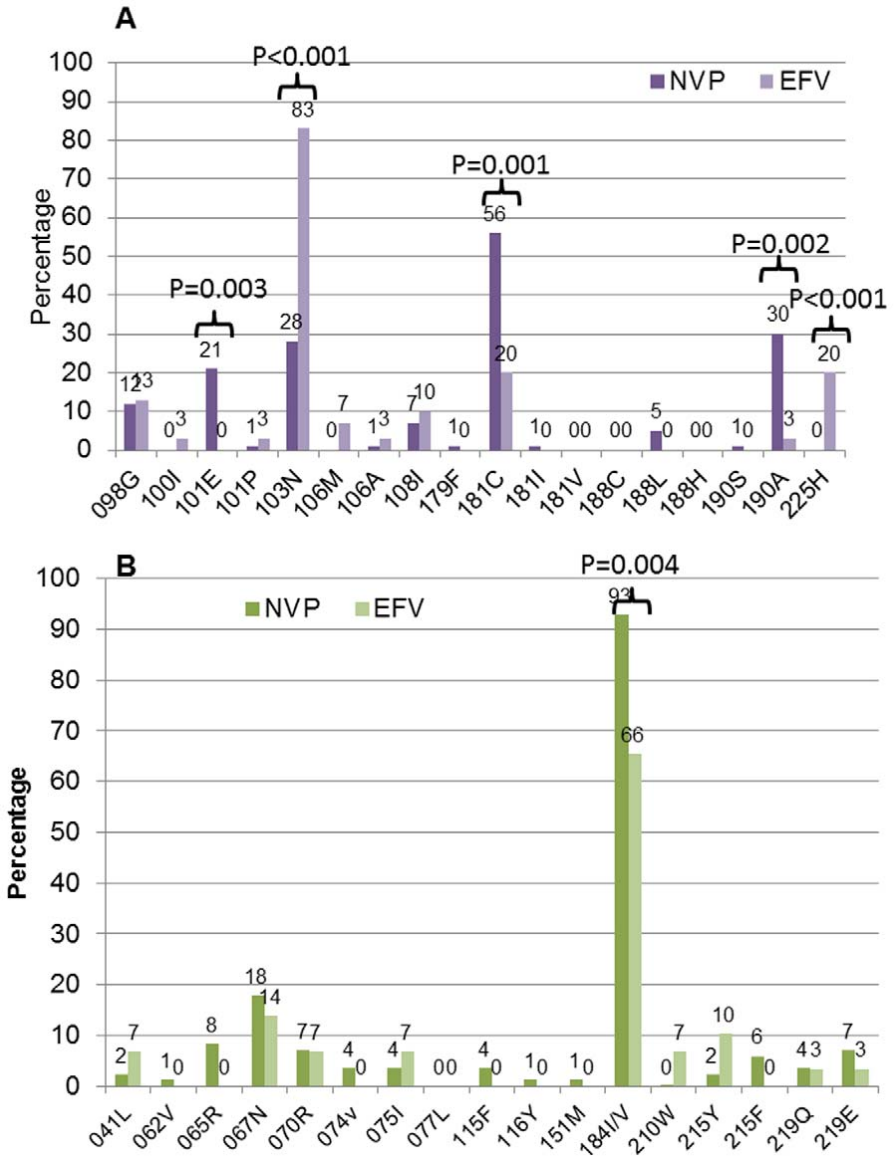
Frequency of resistance mutations observed in subjects failing nevirapine- or efavirenz (EFV)-based treatment. (a) NNRTI resistance mutations and (b) NRTI resistance mutations observed in 89 subjects failing nevirapine- and 30 failing efavirenz (EFV)-based treatment.

The frequency of NNRTI resistance mutations among the nevirapine- and efavirenz-based treatment groups is shown in Figure 1A and that of NRTI resistance mutations in Figure 1B. In the NVP-based treatment group, 100% had virus with one or more NNRTI mutations: Y181C/I was present in 56% (18% as the sole mutation), G190A/S in 30% (4%) and K103N in 28% (18%).

Among the efavirenz-based group, 93% had virus with one or more NNRTI mutation: K103N was present in 83% (32% as the sole mutation). One fourth of samples had Y181C/I or G190A mutations.

The K103N (P<0.001) and P225H (P<0.001) mutations were each significantly more common and Y181C/I (P = 0.001), G190A (P = 0.002) and K101Q/E (P = 0.003) were each significantly less common among subjects failing efavirenz-based treatment as compared to those failing nevirapine-based treatment.

The most prevalent NRTI mutations in both NNRTI groups were M184V/I (93% in nevirapine and 66% in efavirenz). Four percent (4 of 89) of the nevirapine-based treatment group and 32% of the efavirenz-based treatment group had virus with no NRTI resistance mutations.

Of the nine K65R mutations observed, all were found in patients on NVP-based treatment, 6 among patients on TDF and 3 among those on d4T.

### Number of NRTI and NNRTI resistance mutations

The number of NRTI and NNRTI resistance mutations per subject was not significantly different between the efavirenz and nevirapine study subjects. However, tenofovir, when used in the backbone (in comparison to d4T), was found associated with lower occurrence of NRTI mutations when combined with efavirenz (OR = 0. 58, 90%-CI = [0.15,1.46], posterior probability (PP)[OR<1] = 87%) and higher occurrence of both NRTI (OR = 1.58, 90%-CI = [0.95,2.37], PP[OR>1] = 93%) and NNRTI mutations (OR = 1.55, 90%-CI = [0.94,2.12], PP[OR>1] = 93%) when combined with nevirapine. Zidovudine backbone (also in comparison to d4T) was found associated with higher occurrence of NRTI mutations when combined with efavirenz (OR = 2.80, 90%-CI = [0.97,6.35], PP[OR>1] = 94%).

Longer duration of failure was associated with more frequent occurrence of NRTI mutations in patients on nevirapine-based treatment, with about 5% additional risk of any NRTI resistance mutation per additional month on virologic failure (OR = 1.05, 90%-CI = [1.02,1.08], PP[OR>1] = 99.9%). On average, this corresponds to one new NRTI resistance mutation every 20 additional months spent in failure.

No strong evidence was found for the effect of viral load at genotyping. There was also no effect of time to virologic failure.

### Model-based analysis, mutation by mutation

Viral load in the first sample after failure, failure duration and NRTI backbone were the most predictive variables for this analysis. These variables were selected by the statistical model-building procedure, which systematically favored random effects instead of fixed effects models, and were therefore included in the final model as mutation-specific variables. Time to failure was not selected by the model-building procedure and was hence excluded from the analysis.

**Figure 2:**
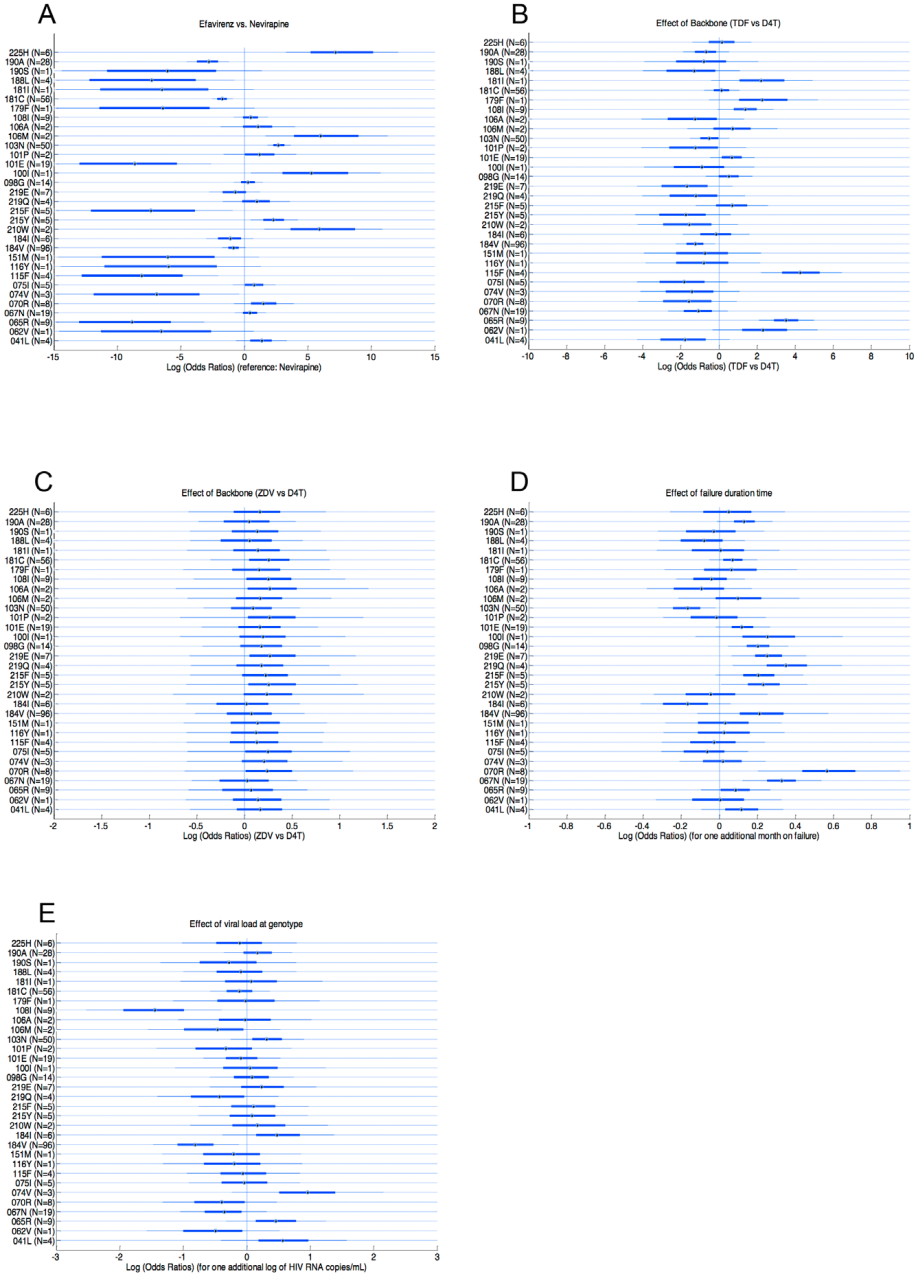
Posterior distributions of the log odds ratios of analyzed parameters for each resistance mutation. Median posterior distributions, 50%- and 90%-credibility intervals are represented. Distributions are based on 1000 simulations using WinBUGS software. In parentheses are reported the number of patients for which the mutation was observed. A. Distributions of the log odds ratios of efavirenz vs. nevirapine-based HAART. Points to the left of the zero vertical line indicate a greater frequency of the indicated mutation in NVP-based regimens, and points to the right of the zero vertical line in EFV-based regimens. B. Distributions of the log odds ratios of tenofovir (TDF) vs. d4T-based backbone. Points to the left of the zero vertical line indicate a greater frequency of the indicated mutation in d4T-based regimens, and points to the right of the zero vertical line in TDF-based regimens. C. Distributions of the log odds ratios of zidovudine (ZDV) vs. d4T-based backbone. Points to the left of the zero vertical line indicate a greater frequency of the indicated mutation in d4T-based regimens, and points to the right of the zero vertical line in ZDV-based regimens. D. Effect of duration of failure. Distributions of the log odds ratios of one additional month spent on failure. Points to the left of the zero vertical line indicate a greater frequency of the indicated mutation when failure is one-month shorter, and points to the right when failure is one-month longer. E. Effect of viral load at genotype. Distributions of the log odds ratios of each resistance mutation for one additional log of HIV RNA copy/mL. Points to the left of the zero vertical line indicate a greater frequency of the indicated mutation when viral load at genotype is one log lower, and points to the right when viral load is one log higher.

When adjusted for the effects of failure duration, viral load and NRTI backbone, the analysis, presented in Figure 2A, showed that the use of a nevirapine-based regimen was associated with significantly increased risks of mutations G190A (the posterior probability of G190A occurring on a nevirapine-based regimen or PP[OR<1] was >99%), Y188L (PP[OR<1] = 97.5%), Y181C (PP[OR<1]>99%), K101E (PP[OR<1]>99%), Y115F (PP[OR<1]>99%) and K65R PP[OR<1]>99%) as compared with an efavirenz-based treatment. A trend toward association with M184V (PP[OR<1] = 92%) was also observed. Conversely, the use of an efavirenz-based regimen was associated with significantly increased risks of the mutations P225H (PP[OR>1]>99%), V106M (PP[OR>1]>99%), K103N (PP[OR>1]>99%), L100I (PP[OR>1] = 97%), T215Y (PP[OR>1] = 97.5%) and L210W (PP[OR>1]>99%). The last two mutations belong to the TAM-1 pathway.

The NRTI backbone was found to influence the emergence of some resistance mutations. More specifically, TDF, when compared with d4T, was found strongly associated with greater risk of mutations Y115F and K65R (PP[OR>1]>99%), and, with less clear evidence (PP[OR>1]>92) with greater risk of mutations Y181I, V179F, and A62V (Figure 2B). TDF was also associated with lower risk of mutation M184V (PP[OR<1] = 99%) and with less significance, of mutation V75I (PP[OR<1] = 89%). When ZDV was compared to d4T (Figure 2C), no significant associations were seen, but in each of the 32 mutations investigated, ZDV posed a higher risk of mutations, with posterior probabilities up to 80% (Supporting Information S1).

Longer duration of virologic failure was found significantly associated with higher risk of all TAM-2 mutations (D67N, K70R, K219Q/E, T215F) and the T215Y mutation with ORs ranging from 1.25 to 1.84 and PP[OR>1]>98% (Figure 2D). Longer duration of failure was also significantly associated with higher risk of M184V (PP[OR>1] = 94%) and NNRTI mutations G190A (PP[OR>1] = 95%), K101E (PP[OR>1] = 94%) and A98G (PP[OR>1] = 99%). Occurrence of K103N was associated with shorter duration of failure (PP[OR<1] = 97%).

Low to moderate evidence (PP[OR>1]<80%) was found for association of viral load at genotyping with higher risk of mutations, with the single exception of 74V (OR = 3.42, 90%-CI = [0. 97,8.61], PP[OR>1] = 94%). Conversely, lower viral load at genotyping was associated with occurrence of mutation M184V (OR = 0.48, 90%-CI = [0. .22,0.86], PP[OR<1] = 98%) and V108I (OR = 0.28, 90%-CI = [0. 06,0.67], PP[OR<1] = 99%).

### Cluster analysis

**Figure 3:**
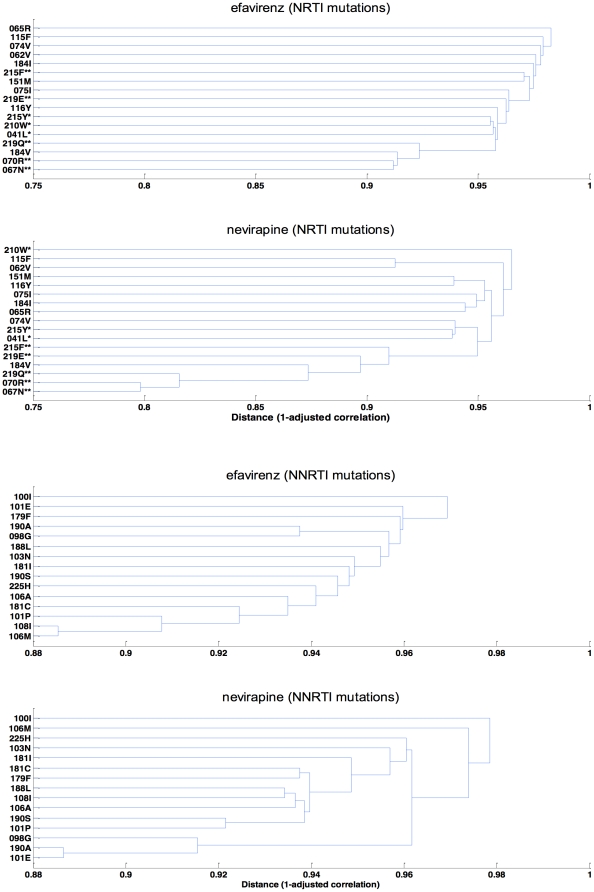
Dendrograms showing correlations between resistance mutations for both nevirapine- and efavirenz-based HAART groups. The distance between clusters is defined as 1-Pearson correlation adjusted for backbone treatment, and failure duration. Smaller distance indicates greater correlation between mutations (clustering). A. Correlations between NNRTI resistance mutations. B. Correlations between NRTI resistance mutations. Asterisks (*) and (**) indicate respectively TAM-1 and TAM-2 mutations.

The cluster analysis was performed to identify patterns of mutation occurrence based on the correlation structure of the data. The outcomes of the cluster analysis are displayed as dendrograms in Figure 3. Overall, both NNRTI and NRTI resistance mutations appear to be substantially less inter-correlated for efavirenz-based treatment as compared with nevirapine-based treatment.

In both efavirenz and nevirapine groups, inter-correlations were weaker for NNRTI (Figure 3A) than for NRTI mutations (Figure 3B). The cluster D67N-K70R-K219Q-M184V, the first three of which are in the TAM-2 pathway, contained the NRTI mutations most likely to occur together in both treatment groups (Figure 3B). With NVP, the next two mutations, K219E and T215F, complete the TAM-2 cluster, whereas with EFV, the three TAM-1 mutations (M41L, L210W, and T215Y) appear next, along with the K219E from the TAM-2 pathway.

### Predicted drug resistance patterns during ARV failure

**Figure 4:**
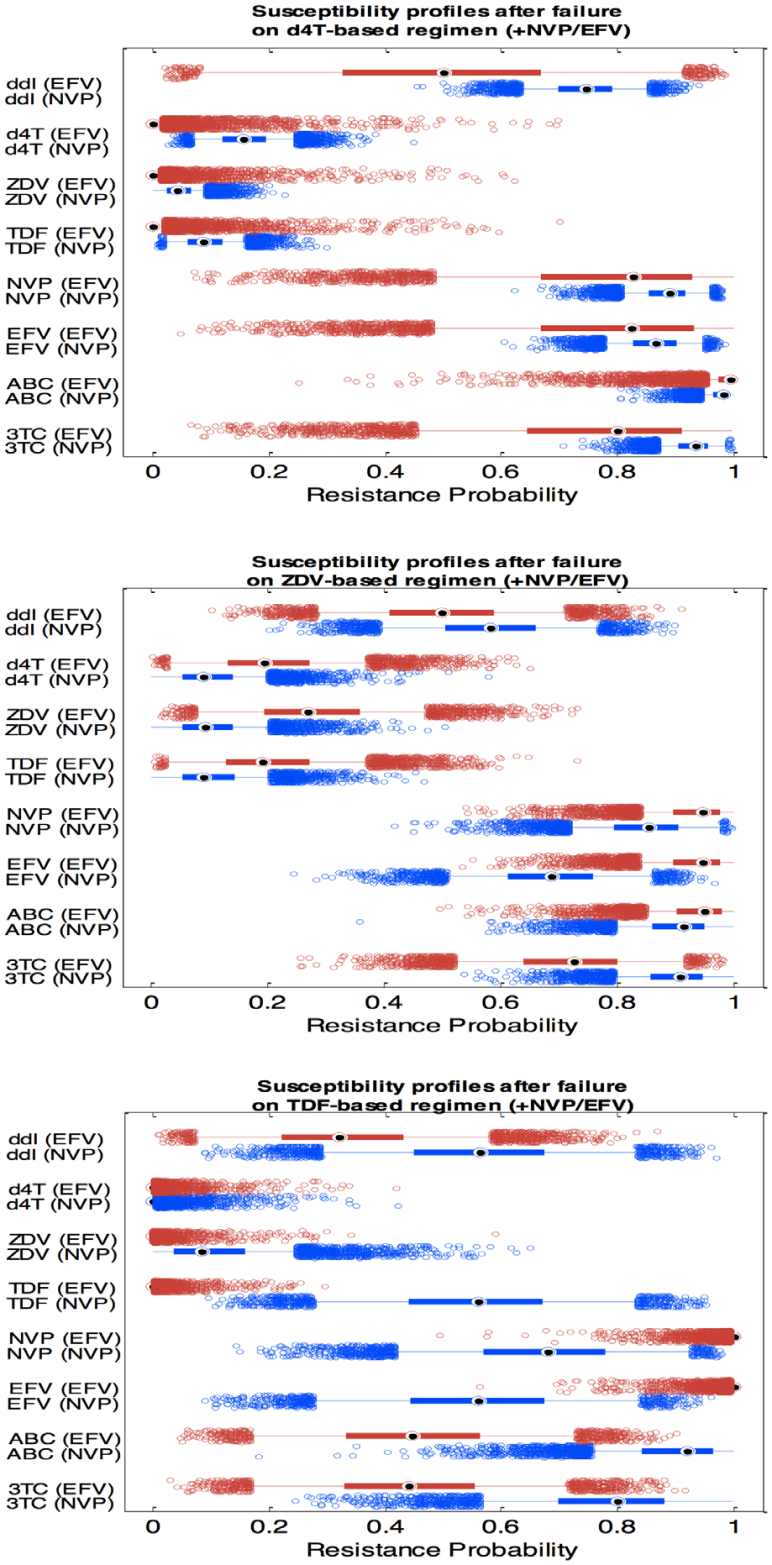
Posterior distribution estimates of the probabilities to belong to resistant subpopulation after virologic failure. Probabilities of resistance to 3TC, ABC, EFV, NVP, TDF, ZDV, d4T and ddI are shown. Based on a simulated sample (n = 5000), boxplots display median (solid square with circle), 25th and 75th percentiles (wide horizontal line), 90% credibility interval (narrow line), and outliers (small circles) for nevirapine (blue boxplots) vs. efavirenz (red boxplots). A. Failure on treatment with d4T-based backbone, B. Failure on treatment with zidovudine-based backbone. C. Failure on treatment with tenofovir-based backbone.

Based on the measured sequences, the best-predicted phenotype and a resistance probability score for each drug that might be used in subsequent treatment were derived using the Geno2Pheno system [20]. The NRTI backbone (observed at time of failure) was the only factor, besides NNRTI choice, influencing the resistance patterns observed on NVP- versus EFV-based regimens, as evidenced by the model-building process. Figure 4 displays boxplots showing the WinBUGS-generated posterior distributions of resistance probabilities for each drug, comparing nevirapine-based and efavirenz-based HAART, and with NRTI backbones containing d4T, ZDV or TDF at the time of failure.

Among those in failure while receiving a d4T-containing backbone (Figure 4A), patients' viruses were predicted to be resistant to abacavir (ABC) and 3TC, while they remained susceptible to d4T, ZDV and TDF whether they had been on NVP or EFV Likewise, they were quite uniformly resistant to both NNRTI's (Supporting Information S2).

With a ZDV-based backbone (Figure 4B), the program predicted marginally lower resistance to d4T, ZDV and TDF in those receiving NVP than in those on EFV-based regimens. Interestingly some susceptibility to EFV persisted in those who had failed on NVP (Supporting Information S3). With TDF, the situation showed sharper contrasts (Figure 4C). Those failing on EFV retained full susceptibility to TDF (zero resistance probability), whereas the nevirapine-based HAART group had a 55% chance to be resistant (Supporting Information S4). Likewise, some susceptibility was predicted for ABC and 3TC in the EFV-based group, but little was seen in those failing on NVP. In contrast, moderate susceptibility to EFV and, to a lesser extent, NVP was retained by those who failed while on NVP, whereas there was essentially complete resistance to both drugs in those on EFV-based regimens.

## Discussion

This study, using standard and model-based Bayesian analytic methods, presents the first detailed comparison of the ARV resistance patterns found during virologic failure for NVP- vs. EFV-based combination regimens in a group of subjects infected with CRF01_AE strains of HIV-1. Our findings emphasize differences and similarities from the patterns seen during failure in patients infected with other subtypes.

The clearest differences found between treatment groups were in specific resistance mutations or clusters rather than in overall total numbers of mutations. Our analysis offers strong evidence that, in contrast to the mutation patterns published for other subtypes [12], [13], [14], [15], [21], individuals infected with CRF01_AE and experiencing virologic failure while receiving NVP-based HAART favor TAM-2 resistance pathways (K70R, D67N, T215F, K219Q/E) rather than TAM-1 (M41L, L210W, T215Y), whereas those receiving regimens containing EFV appear to select both TAM-2 and TAM-1 pathways (Figures 1B, 2A and 3B).

As suggested by previous studies of mainly Subtype B viruses [10], [22], [23], [24], [25], [26], and as shown in both the raw percentages (Figure 1A and 1B) and the adjusted Bayesian analysis (Figure 2A), mutations 101E, 181C and 190A were preferentially selected by nevirapine, while 103N, 106M, and 225H were preferentially selected by efavirenz. Wallis, in her study of subtype C, found similar differential distributions of 101E, 181C, 190A and 106M but found almost equal proportions of 103N and 225H mutations selected by EFV and NVP [11]. Reuman et al. also found that K103N, V106M and P225H were among the 16 NNRTI mutations preferentially selected by EFV and K101E, Y181C and G190A among the 12 mutations preferentially selected by NVP. However, in their analysis of covariation of NNRTI resistance mutations, the K103N-P225H pair as well the Y181C-G190A and K101E-Y181C pairs significantly covaried in sequences from individuals experiencing EFV while the pair V108I-Y181C covaried in NVP group and the pair K101E-G190E covaried in both groups [10]. In our study, the pair 101E-190A of NNRTI mutations was closely correlated only in the nevirapine group.

Less expected was our finding that the major NRTI mutations M184V and K65R were preferentially selected in the presence of nevirapine (Figures 1B and 2A), while TAM-1 mutations were almost never selected (only in 2% of patients treated by nevirapine). In contrast, TAM-1 215Y and 210W were preferentially selected in the presence of efavirenz.

The NRTI backbone was shown to influence the resistance patterns (see Figures 2B and 2C). Our model-based analysis suggested that tenofovir, in addition to selecting K65R, also strongly selected Y115F (Figure 2B), a mutation rarely observed in other subtypes, and then only with the use of a triple-NRTI regimen [27], [28], [29]. In contrast, we confirmed the observation that K65R has an antagonistic effect with TAMs [30], since only 1 of 9 patients in our study had both 65R and one TAM. Also we found that tenofovir use was associated with a significantly lower rate of M184V mutation than d4T, illustrated in Figure 2B, while this was not observed in the 903 study which evaluated the efficacy and safety of tenofovir vs stavudine when combined with 3TC and Efavirenz in antiretroviral-naive patients [31]. The trend (seen in 26 of 32 mutations analyzed) towards higher rates of both NRTI and NNRTI mutations observed with zidovudine in comparison to d4T (Figure 2C) is consistent with observations by Wallis in South Africa [11] and Bocket in France [12].

It is likely that the accumulation of mutations resulting in drug resistance follows specific pathways rather than random sequences. Duration of virologic failure may be associated with the order of mutation occurrence, and therefore with the timing of some mutations. Our analysis, shown in Figure 2D, confirmed that the M184I mutation occurs before M184V, suggested that K103N is an early mutation, and showed clearly that all the TAM-2 pathway mutations, as well as 215Y, occur as time spent on virologic failure increases.

Overall, high viral load at genotyping was not seen to favor mutation occurrence after failure. Conversely, the clear negative association of viral load at genotyping and occurrence of mutations M184V and V108I can be interpreted as an effect of these mutations on viral fitness. Impairment of HIV fitness in viruses containing the 184V mutation is well known [32]. There is one report of lower viral loads with the 108I mutation, and this analysis confirms that finding [33]. The actual fitness of virus with this mutation has not been investigated.

In our analysis of resistance and susceptibility to a range of available drugs, failure on efavirenz-based HAART seemed to impair any further use of efavirenz and nevirapine, while this was not systematically the case with nevirapine (Figures 4A–C). The Geno2Pheno software analysis predicted that resistant virus selected by nevirapine may still be susceptible to efavirenz and even, although to a lesser extent, to nevirapine. EFV sensitivity is likely due to the Y181C mutation, as reported by other groups [34], [35]. The persistent nevirapine susceptibility implies the possibility that nevirapine may be successfully recycled (perhaps after the elapse of some time) in resource-constrained environments. Overall it appears that, in terms of the salvage treatment options, zidovudine should preferably be associated with nevirapine rather than with efavirenz, whereas tenofovir should better be associated with efavirenz, consistent with the current DHHS guidelines [36]. The synergistic association of tenofovir and efavirenz is also supported by the fact that efavirenz was shown to protect from K65R and Y115F.

One obvious limitation of this analysis is the cross-sectional nature of the data and methods used. Only one genotype assessment was used per patient so that the dynamics and timing of resistance mutations could not be investigated. The clusters identified could therefore not be imputed to some time ordering.

Although the genotype data were of good and consistent quality as they originated from a single quality-controlled laboratory, nevertheless, some missing information could alter the precision and accuracy of some estimates. In addition, the Geno2Pheno predictions were analyzed as raw data, without accounting for possible small numbers and any uncertainty in the model since it was not available on the Max-Planck-Institute Informatik platform.

Finally it should be re-emphasized that our comparative assessment was made on subjects who had failed their first-line treatment. Such a study design is less robust than a prospective, randomized design would have been, but focuses only on events at virologic failure. To complete and deepen this assessment, a similar model-based assessment could be developed on the population at start of treatment, integrating failure rate and timing of failure. Such an analysis would be strengthened by the use of longitudinal data and methods which could provide a clearer view of resistance mutation pathways over time to support optimal monitoring and medical decisions throughout treatment.

Nevertheless, our model-based evaluation allowed the comparative assessment of resistance mutation patterns between nevirapine-based and efavirenz-based treatment groups, disentangling the concurrent effects of NRTI backbone and other factors and accounting for correlations between mutations. The Bayesian tools enabled the statistical inference of such models and provided comprehensive outputs and measures of uncertainty attached to the results. The analysis not only confirmed well-established patterns already observed in other studies with other subtypes [8], [10], [13], [22], [23], [24], [25], [26], [37], but also pointed at less known or new features to be considered for optimal treatment and future research.

## Materials and Methods

### Study population

The study population includes 139 HIV-infected adults enrolled in the PHPT-GFATM cohort treatment program supported by the Thai Ministry of Public Health and the Global Fund to Fight AIDS, Tuberculosis and Malaria (ClinicalTrials.gov Identifier: NCT00433030). The larger cohort has been described [Fregonese F, Collins I, Jourdain G, Le Coeur S, Cressey T, Ngo-Giang-Huong N, Banchongkit S, Chutanunta A, Techapornroong M, Lallemant M, for the Program for HIV Prevention and Treatment (PHPT) study group. Survival of HIV-infected Adults Starting HAART in Thailand: Risk Factors for Early and Long term Mortality. 18^th^ Conference on Retroviruses and Opportunistic Infections, Boston, MA, USA, 2011. Abstract 561]. Subjects were included in this analysis if they had experienced virologic failure while on first-line nevirapine- or efavirenz-based HAART and had genotypic resistance testing before switching to second line HAART.

At therapy initiation, study subjects were antiretroviral-naïve except for prophylaxis of mother-to-child transmission of HIV. HIV RNA levels and CD4 cell counts were measured at treatment initiation, three months, six months and every six months thereafter. Virologic failure was defined as HIV RNA concentration greater than 500 copies/mL after 6 months of HAART. For Table 1, the date of virologic failure was defined as the midpoint between the last viral load <500 copies/mL and the first viral load >500 copies/mL. In the model-based analysis, the duration of virologic failure was estimated taking into account the dates of the last viral load <500 copies/mL and the first viral load >500 copies/mL as well as the frequency of blood sampling.

### Measurement of plasma HIV-1 RNA

Plasma HIV-1 RNA levels were quantified using the standard (limit of detection, 400 copies/mL) or the ultrasensitive (limit of detection, 50 copies/mL) protocol of the Cobas Amplicor HIV-1 Monitor RNA test, version 1.5 (Roche Molecular Systems Inc., Branchburg, USA).

### Genotypic resistance testing

HIV-1 Resistance testing for the RT gene was performed using the ViroSeq HIV-1 Genotyping system (Celera Diagnostics, Alameda, USA) according to the manufacturer's instructions or using the consensus technique of the Agence Nationale de Recherches sur le SIDA (AC11 Resistance Study Group PCR and Sequencing Procedures http://www.hivfrenchresistance.org/ANRS-procedures.pdf). The first round of nested PCR was performed on extracted RNA, with the Kit Titan One tube (Roche Diagnostics) and the set of MJ3 and MJ4 primers. The second round PCR used the set of A35 and NEI135 primers. PCR products were purified using the QIAQUICK Purification PCR kit (QIAGEN). In both techniques, sequencing products were then submitted onto the automated genetic analyzer 3100 (Applied Biosystems, Foster city, CA, USA). Sequences were aligned using the Viroseq or Seqscape softwares (Applied Biosystems). RT mutations were identified from the International AIDS Society USA Drug (IAS-USA) mutation tables, spring 2008 (http://www.iasusa.org/resistance_mutations): M41L, K65R, D67N, insertion 69, K70R/E, L74I/V, L100I, K103N, V106A/M, V108I, Q151M, Y181I/C, M184V, Y188C/L, G190A/S, L210W, T215Y/F, K219Q/E, P225H and M230L. The only additions to this list since that time are K101H, E138A, and M230L, all etravirine-associated changes and K101P associated with resistance to NVP, EFV and etravirine. GenBank accession numbers are HQ996409 to 996529.

### Descriptive statistics and demographics

Demographic, laboratory, and clinical characteristics at the time of genotyping such as CD4 count, HIV RNA, CDC stage, and RT mutation frequencies were compared between the NVP- and EFV-based HAART groups. Categorical variables were compared using Chi-square or Fisher's exact tests; reported P-values were two-tailed. Continuous variables were compared using the Wilcoxon rank-sum test.

### Comparison of resistance mutation counts at failure

The total number of NRTI or NNRTI resistance mutations was compared between the nevirapine and efavirenz groups. In order to investigate the possible effect of NRTI or NNRTI mutations, a bivariate binomial-logistic model was fitted to the numbers of NRTI and NNRTI mutations, accounting for their correlation using a patient-specific random effect. The logistic regression component permitted an adjustment for the treatment used (nevirapine vs. efavirenz and NRTI backbone), failure duration, time to failure, and the viral load at genotyping.

### Mutation-by-mutation analysis

A multivariate logistic regression model was fitted to any NRTI or NNRTI mutations observed. Covariates considered were NNRTI treatment (nevirapine or efavirenz), backbone drug (TDF vs. ZDV vs. d4T), failure duration, viral load at failure, and time to failure. Those covariates were assumed to have either mutation-specific effects (random or fixed) or constant effects across mutations. These choices were based on the performance of the fitting algorithms (robust convergence) and based on statistical model selection criteria (Deviance Information Criteria [38], favoring models which can better reproduce the data observed with most parsimonious parameterization. Odds ratios (ORs) were derived for each of these factors.

### Analysis of correlations between resistance mutations (cluster analysis)

We performed cluster analysis in order to analyze the multivariate correlation patterns between resistance mutations observed. The model described in the previous paragraph allowed the derivation of a correlation matrix of all NRTI and NNRTI mutations, adjusted for backbone, viral load and duration of failure effects. Based on this adjustment, a distance, defined as (1 - correlation), was used to describe the clustering of mutations. The nearest neighbor algorithm was used as linkage method to determine in what order clusters may join with each other. Results are displayed for both NRTI and NNRTI mutations using a correlation tree or dendrogram, which lists all mutations and indicates at what level of similarity (or correlation) any two clusters joined together. The main features of dendrograms for nevirapine-based and efavirenz-based treatments were then described and compared.

### Phenotype inference

The nucleotide sequences of the region coding for the reverse transcriptase were submitted to the web-based user interface of the Max-Planck-Institute Informatik Geno2Pheno website [20]. Based on the alignment of the uploaded genotype sequence with the HXB2 reference and on machine learning approaches, the Geno2Pheno system derives the best-predicted phenotype and a resistance probability score for a list of drugs. Probability scores were made available for each viral sequence for the following antiretroviral drugs: the NRTIs ZDV, ddI, d4T, 3TC, emtricitabine (FTC), ABC, and TDF; and the NNRTIs NVP and EFV.

### Comparative assessment of drug resistance

For the purpose of modeling, the distributions of Geno2Pheno-predicted resistance probabilities were dichotomized. The resulting binary data were analyzed by a Bernoulli/logistic regression to compare nevirapine- versus efavirenz-based HAART, with adjustment for NRTI backbone at failure and with all significant or relevant covariates included in the model-building phase. Once the model was fitted to the data, the predictive drug resistance distributions were compared between efavirenz and nevirapine, and between the d4T-, ZDV- and TDF-containing backbones.

### Bayesian statistical inference and model-building

Bayesian inference was used to fit the statistical models described above [39]. Briefly, in this framework, prior information about the quantities of interest was combined with the observed data to derive a posterior distribution on these quantities, using Monte Carlo Markov chains algorithms. In all analyses, only non-informative priors were used. Bayesian inference is appropriate for mixed-effect and/or non linear models like the ones used, especially in the case of small sample sizes [39], [40]. The 90%-credibility interval (CI) attached to point estimates are presented and can be directly interpreted as the range within which there is 90% chance that the quantity of interest lies.

Point estimates were reported as mean posterior estimates. For odds ratios estimates, their (posterior) probability to be greater than 1 (PP[OR>1]) or lower than 1 (PP[OR<1]) was reported in addition to credibility intervals. To visualize posterior distributions, posterior medians, inter-quartile ranges, and 90%-credibility intervals were presented as boxplots using *ad hoc* routines coded in Matlab Release 14.

The model-building phase was consistently implemented as follows. First, the model was fitted to the data without any covariate. Then, the resulting estimates were used as initial values. The WinBUGS software (version 1.4, [41]) was used for both the assessment of the model-building and the final models.

## Supporting Information

Supporting Information S1 Posterior probabilities [OR>1] for each mutation. Posterior probabilities are provided for each NNRTI and NRTI resistance mutation.
(DOC)

Supporting Information S2 Resistance probabilities with a d4T backbone. Probabilities of virus to be resistant to 3TC, ABC, EFV, NVP, TDF, d4T and ddI (95% confidence interval) among patients failing a d4T-containing backbone in combination with NVP or EFV.
(DOC)

Supporting Information S3 Resistance probabilities with a ZDV backbone. Probabilities of virus to be resistant to 3TC, ABC, EFV, NVP, TDF, d4T and ddI (95% confidence interval) among patients failing a ZDV-containing backbone in combination with NVP or EFV.
(DOC)

Supporting Information S4 Resistance probabilities with a TDF backbone. Probabilities of virus to be resistant to 3TC, ABC, EFV, NVP, TDF, d4T and ddI (95% confidence interval) among patients failing a TDF-containing backbone in combination with NVP or EFV.
(DOC)

## References

[1] World Health Organization (2010). Antiretroviral therapy for HIV infection in adults and adolescents: recommendations for a public health approach. – 2010 rev.

[2] Deshpande A, Jeannot AC, Schrive MH, Wittkop L, Pinson P (2010). Analysis of RT sequences of subtype C HIV-type 1 isolates from indian patients at failure of a first-line treatment according to clinical and/or immunological WHO guidelines.. AIDS Res Hum Retroviruses.

[3] Hawkins CA, Chaplin B, Idoko J, Ekong E, Adewole I (2009). Clinical and genotypic findings in HIV-infected patients with the K65R mutation failing first-line antiretroviral therapy in Nigeria.. J Acquir Immune Defic Syndr.

[4] Hosseinipour MC, van Oosterhout JJ, Weigel R, Phiri S, Kamwendo D (2009). The public health approach to identify antiretroviral therapy failure: high-level nucleoside reverse transcriptase inhibitor resistance among Malawians failing first-line antiretroviral therapy.. AIDS.

[5] Manosuthi W, Butler DM, Chantratita W, Sukasem C, Richman DD (2010). Patients infected with HIV type 1 subtype CRF01_AE and failing first-line nevirapine- and efavirenz-based regimens demonstrate considerable cross-resistance to etravirine.. AIDS Res Hum Retroviruses.

[6] Marconi VC, Sunpath H, Lu Z, Gordon M, Koranteng-Apeagyei K (2008). Prevalence of HIV-1 drug resistance after failure of a first highly active antiretroviral therapy regimen in KwaZulu Natal, South Africa.. Clin Infect Dis.

[7] Orrell C, Walensky RP, Losina E, Pitt J, Freedberg KA (2009). HIV type-1 clade C resistance genotypes in treatment-naive patients and after first virological failure in a large community antiretroviral therapy programme.. Antivir Ther.

[8] Sungkanuparph S, Manosuthi W, Kiertiburanakul S, Piyavong B, Chumpathat N (2007). Options for a second-line antiretroviral regimen for HIV type 1-infected patients whose initial regimen of a fixed-dose combination of stavudine, lamivudine, and nevirapine fails.. Clin Infect Dis.

[9] Soria A, Porten K, Fampou-Toundji JC, Galli L, Mougnutou R (2009). Resistance profiles after different periods of exposure to a first-line antiretroviral regimen in a Cameroonian cohort of HIV type-1-infected patients.. Antivir Ther.

[10] Reuman EC, Rhee SY, Holmes SP, Shafer RW (2010). Constrained patterns of covariation and clustering of HIV-1 non-nucleoside reverse transcriptase inhibitor resistance mutations.. J Antimicrob Chemother.

[11] Wallis CL, Mellors JW, Venter WD, Sanne I, Stevens W (2010). Varied patterns of HIV-1 drug resistance on failing first-line antiretroviral therapy in South Africa.. J Acquir Immune Defic Syndr.

[12] Bocket L, Yazdanpanah Y, Ajana F, Gerard Y, Viget N (2004). Thymidine analogue mutations in antiretroviral-naive HIV-1 patients on triple therapy including either zidovudine or stavudine.. J Antimicrob Chemother.

[13] Kuritzkes DR, Bassett RL, Hazelwood JD, Barrett H, Rhodes RA (2004). Rate of thymidine analogue resistance mutation accumulation with zidovudine- or stavudine-based regimens.. J Acquir Immune Defic Syndr.

[14] Marcelin AG, Delaugerre C, Wirden M, Viegas P, Simon A (2004). Thymidine analogue reverse transcriptase inhibitors resistance mutations profiles and association to other nucleoside reverse transcriptase inhibitors resistance mutations observed in the context of virological failure.. J Med Virol.

[15] Novitsky V, Wester CW, DeGruttola V, Bussmann H, Gaseitsiwe S (2007). The reverse transcriptase 67N 70R 215Y genotype is the predominant TAM pathway associated with virologic failure among HIV type 1C-infected adults treated with ZDV/ddI-containing HAART in southern Africa.. AIDS Res Hum Retroviruses.

[16] Ariyoshi K, Matsuda M, Miura H, Tateishi S, Yamada K (2003). Patterns of point mutations associated with antiretroviral drug treatment failure in CRF01_AE (subtype E) infection differ from subtype B infection.. J Acquir Immune Defic Syndr.

[17] Sukasem C, Churdboonchart V, Sukeepaisarncharoen W, Piroj W, Inwisai T (2008). Genotypic resistance profiles in antiretroviral-naive HIV-1 infections before and after initiation of first-line HAART: impact of polymorphism on resistance to therapy.. Int J Antimicrob Agents.

[18] Sutthent R, Arworn D, Kaoriangudom S, Chokphaibulkit K, Chaisilwatana P (2005). HIV-1 drug resistance in Thailand: before and after National Access to Antiretroviral Program.. J Clin Virol.

[19] National AIDS Prevention and Alleviation Committee (2010). UNGASS COUNTRY PROGRESS REPORT THAILAND..

[20] Beerenwinkel N, Daumer M, Oette M, Korn K, Hoffmann D (2003). Geno2pheno: Estimating phenotypic drug resistance from HIV-1 genotypes.. Nucleic Acids Res.

[21] Cozzi-Lepri A, Phillips AN, Martinez-Picado J, Monforte A, Katlama C (2009). Rate of accumulation of thymidine analogue mutations in patients continuing to receive virologically failing regimens containing zidovudine or stavudine: implications for antiretroviral therapy programs in resource-limited settings.. J Infect Dis.

[22] Bacheler LT, Anton ED, Kudish P, Baker D, Bunville J (2000). Human immunodeficiency virus type 1 mutations selected in patients failing efavirenz combination therapy.. Antimicrob Agents Chemother.

[23] Bannister WP, Ruiz L, Cozzi-Lepri A, Mocroft A, Kirk O (2008). Comparison of genotypic resistance profiles and virological response between patients starting nevirapine and efavirenz in EuroSIDA.. AIDS.

[24] Deforche K, Camacho RJ, Grossman Z, Soares MA, Van Laethem K (2008). Bayesian network analyses of resistance pathways against efavirenz and nevirapine.. AIDS.

[25] Delaugerre C, Rohban R, Simon A, Mouroux M, Tricot C (2001). Resistance profile and cross-resistance of HIV-1 among patients failing a non-nucleoside reverse transcriptase inhibitor-containing regimen.. J Med Virol.

[26] Richman DD, Havlir D, Corbeil J, Looney D, Ignacio C (1994). Nevirapine resistance mutations of human immunodeficiency virus type 1 selected during therapy.. J Virol.

[27] Lyagoba F, Dunn DT, Pillay D, Kityo C, Robertson V (2010). Evolution of Drug Resistance During 48 Weeks of Zidovudine/Lamivudine/Tenofovir in the Absence of Real-Time Viral Load Monitoring.. J Acquir Immune Defic Syndr.

[28] Rey D, Krebs M, Partisani M, Hess G, Cheneau C (2006). Virologic response of zidovudine, lamivudine, and tenofovir disoproxil fumarate combination in antiretroviral-naive HIV-1-infected patients.. J Acquir Immune Defic Syndr.

[29] Ross L, Elion R, Lanier R, Dejesus E, Cohen C (2009). Modulation of K65R selection by zidovudine inclusion: analysis of HIV resistance selection in subjects with virologic failure receiving once-daily abacavir/lamivudine/zidovudine and tenofovir DF (study COL40263).. AIDS Res Hum Retroviruses.

[30] Parikh UM, Bacheler L, Koontz D, Mellors JW (2006). The K65R mutation in human immunodeficiency virus type 1 reverse transcriptase exhibits bidirectional phenotypic antagonism with thymidine analog mutations.. J Virol.

[31] Gallant JE, Staszewski S, Pozniak AL, DeJesus E, Suleiman JM (2004). Efficacy and safety of tenofovir DF vs stavudine in combination therapy in antiretroviral-naive patients: a 3-year randomized trial.. JAMA.

[32] Back NK, Nijhuis M, Keulen W, Boucher CA, Oude Essink BO (1996). Reduced replication of 3TC-resistant HIV-1 variants in primary cells due to a processivity defect of the reverse transcriptase enzyme.. EMBO J.

[33] Machouf N, Thomas R, Nguyen VK, Trottier B, Boulassel MR (2006). Effects of drug resistance on viral load in patients failing antiretroviral therapy.. J Med Virol.

[34] Casado JL, Moreno A, Hertogs K, Dronda F, Moreno S (2002). Extent and importance of cross-resistance to efavirenz after nevirapine failure.. AIDS Res Hum Retroviruses.

[35] Miller V, de Bethune MP, Kober A, Sturmer M, Hertogs K (1998). Patterns of resistance and cross-resistance to human immunodeficiency virus type 1 reverse transcriptase inhibitors in patients treated with the nonnucleoside reverse transcriptase inhibitor loviride.. Antimicrob Agents Chemother.

[36] (2009). Panel on Antiretroviral Guidelines for Adults and Adolescents. Guidelines for the use of antiretroviral agents in HIV-1-infected adults and adolescents.. Department of Health and Human Services.

[37] Hanna GJ, Johnson VA, Kuritzkes DR, Richman DD, Brown AJ (2000). Patterns of resistance mutations selected by treatment of human immunodeficiency virus type 1 infection with zidovudine, didanosine, and nevirapine.. J Infect Dis.

[38] Spiegelhalter DJ, Best NG, Carlin BP, Van der Linde A (2002). Bayesian measures of model complexity and fit.. J Roy Statist Soc B.

[39] Gelman A, Carlin JB, Stern HS, Rubin DB (2004).

[40] Wakefield J, Smith A, Racine-Poon A (1994). Bayesian Analysis of Linear and NonLinear Population Models.. Applied Stats.

[41] Lunn DJ, Thomas A, Best N, Spiegelhalter D (2000). WinBUGS – a Bayesian modelling framework: concepts, structure and extensibility.. Statistics and Computing.

