# Supplementary material for: Resistance Patterns Selected by Nevirapine vs. Efavirenz in HIV-Infected Patients Failing First-Line Antiretroviral Treatment: A Bayesian Analysis
Source: PLoS One. 2011 Nov 23;6(11):e27427. doi: 10.1371/journal.pone.0027427 (PMC3223170; doi:10.1371/journal.pone.0027427)
Supplement: Supporting Information S1 — Posterior probabilities [OR>1] for each mutation. Posterior probabilities are provided for each NNRTI and NRTI resistance mutation. (DOC) [file pone.0027427.s001.doc]

Supporting Information S1: Posterior probabilities [OR>1] for each mutation

| Mutations | Posterior probabilities [OR>1] |
| --- | --- |
| 041L | 68 |
| 062V | 65 |
| 065R | 57 |
| 067N | 53 |
| 070R | 76 |
| 074V | 72 |
| 075I | 76 |
| 115F | 64 |
| 116Y | 63 |
| 151M | 64 |
| 184V | 60 |
| 184I | 53 |
| 210W | 75 |
| 215Y | 78 |
| 215F | 73 |
| 219Q | 68 |
| 219E | 79 |
| 098G | 71 |
| 100I | 71 |
| 101E | 69 |
| 101P | 78 |
| 103N | 61 |
| 106M | 68 |
| 106A | 78 |
| 108I | 78 |
| 179F | 66 |
| 181C | 80 |
| 181I | 66 |
| 188L | 56 |
| 190S | 64 |
| 190A | 56 |
| 225H | 67 |
