# Supplementary material for: Resistance Patterns Selected by Nevirapine vs. Efavirenz in HIV-Infected Patients Failing First-Line Antiretroviral Treatment: A Bayesian Analysis
Source: PLoS One. 2011 Nov 23;6(11):e27427. doi: 10.1371/journal.pone.0027427 (PMC3223170; doi:10.1371/journal.pone.0027427)
Supplement: Supporting Information S2 — Resistance probabilities with a d4T backbone. Probabilities of virus to be resistant to 3TC, ABC, EFV, NVP, TDF, d4T and ddI (95% confidence interval) among patients failing a d4T-containing backbone in combination with NVP or EFV. (DOC) [file pone.0027427.s002.doc]

Supporting Information S2: Resistance probabilities with a d4T backbone

|  | **d4T backbone with NVP** | | **d4T backbone with EFV** | |
| --- | --- | --- | --- | --- |
|  | Resistance probability | 95% confidence interval | Resistance probability | 95%confidence interval |
| 3TC | 0.9277 | 0.8373-0.9840 | 0.7558 | 0.2917-0.9860 |
| ABC | 0.9743 | 0.9129-0.9991 | 0.9681 | 0.7558-1.0000 |
| EFV | 0.8608 | 0.7446-0.9477 | 0.7767 | 0.3213-0.9943 |
| NVP | 0.8824 | 0.7727-0.9585 | 0.7773 | 0.3266-0.9942 |
| TDF | 0.0928 | 0.0259-0.1908 | 0.0226 | 0.0000-0.2090 |
| ZDV | 0.0476 | 0.0056-0.1264 | 0.0171 | 0.0000- 0.1688 |
| d4T | 0.1594 | 0.0679-0.2757 | 0.022 | 0.0000-0.2107 |
| ddI | 0.7417 | 0.6027-0.8577 | 0.4995 | 0.1088- 0.8985 |
