# Supplementary material for: Resistance Patterns Selected by Nevirapine vs. Efavirenz in HIV-Infected Patients Failing First-Line Antiretroviral Treatment: A Bayesian Analysis
Source: PLoS One. 2011 Nov 23;6(11):e27427. doi: 10.1371/journal.pone.0027427 (PMC3223170; doi:10.1371/journal.pone.0027427)
Supplement: Supporting Information S3 — Resistance probabilities with a ZDV backbone. Probabilities of virus to be resistant to 3TC, ABC, EFV, NVP, TDF, d4T and ddI (95% confidence interval) among patients failing a ZDV-containing backbone in combination with NVP or EFV. (DOC) [file pone.0027427.s003.doc]

Supporting Information S3: Resistance probabilities with a ZDV backbone

|  | **ZDV backbone with NVP** | | **ZDV backbone with EFV** | |
| --- | --- | --- | --- | --- |
|  | Resistance probability | 95% confidence interval | Resistance probability | 95% confidence interval |
| 3TC | 0.8956 | 0.7293-0.9861 | 0.7141 | 0.4632-0.9078 |
| ABC | 0.8975 | 0.7306-0.9866 | 0.9318 | 0.7655-0.9981 |
| EFV | 0.6825 | 0.4658-0.8657 | 0.9263 | 0.7511-0.9978 |
| NVP | 0.8425 | 0.6519-0.9654 | 0.9273 | 0.7467-0.9975 |
| TDF | 0.1039 | 0.0129-0.2628 | 0.2075 | 0.0465-0.4520 |
| ZDV | 0.1056 | 0.0149-0.2744 | 0.281 | 0.0902-0.5344 |
| d4T | 0.1041 | 0.0128-0.2688 | 0.208 | 0.0479-0.4472 |
| ddI | 0.5798 | 0.3601-0.7828 | 0.4999 | 0.2567-0.7480 |
