# Supplementary material for: Resistance Patterns Selected by Nevirapine vs. Efavirenz in HIV-Infected Patients Failing First-Line Antiretroviral Treatment: A Bayesian Analysis
Source: PLoS One. 2011 Nov 23;6(11):e27427. doi: 10.1371/journal.pone.0027427 (PMC3223170; doi:10.1371/journal.pone.0027427)
Supplement: Supporting Information S4 — Resistance probabilities with a TDF backbone. Probabilities of virus to be resistant to 3TC, ABC, EFV, NVP, TDF, d4T and ddI (95% confidence interval) among patients failing a TDF-containing backbone in combination with NVP or EFV. (DOC) [file pone.0027427.s004.doc]

Supporting Information S4: Resistance probabilities with a TDF backbone

|  | **TDF backbone with NVP** | | **TDF backbone with EFV** | |
| --- | --- | --- | --- | --- |
|  | Resistance  probability | 95% confidence interval | Resistance  probability | 95% confidence interval |
| 3TC | 0.7796 | 0.4678-0.9694 | 0.4449 | 0.1610-0.7569 |
| ABC | 0.8898 | 0.6360-0.9962 | 0.4508 | 0.1656-0.7568 |
| EFV | 0.556 | 0.2495-0.8378 | 0.9906 | 0.9073-1.0000 |
| NVP | 0.6667 | 0.3490-0.9155 | 0.9906 | 0.9063-1.0000 |
| TDF | 0.5527 | 0.2356-0.8353 | 0.0056 | 0.0000- 0.0594 |
| ZDV | 0.1111 | 0.0033-0.3676 | 0.0056 | 0.0000- 0.0596 |
| d4T | 0.0099 | 0.0000-0.0980 | 0.0063 | 0.0000- 0.0706 |
| ddI | 0.5579 | 0.2493-0.8430 | 0.3331 | 0.0835- 0.6581 |
